# SIRT1-Dependent Upregulation of Antiglycative Defense in HUVECs Is Essential for Resveratrol Protection against High Glucose Stress

**DOI:** 10.3390/antiox8090346

**Published:** 2019-09-01

**Authors:** Silvano Junior Santini, Valeria Cordone, Mahmut Mijit, Virginio Bignotti, Pierpaolo Aimola, Vincenza Dolo, Stefano Falone, Fernanda Amicarelli

**Affiliations:** 1 Department of Life, Health and Environmental Sciences, University of L’Aquila, 67100 L’Aquila, Italy; 2 Department of Biomedical and Specialist Surgical Sciences, University of Ferrara, 44121 Ferrara, Italy

**Keywords:** oxidative stress, glycative stress, dicarbonyl stress, antioxidant defense, superoxide dismutase, catalase, glyoxalase, EX527, sirtuin 1

## Abstract

Uncontrolled accumulation of methylglyoxal (MG) and reactive oxygen species (ROS) occurs in hyperglycemia-induced endothelial dysfunction associated with diabetes. Resveratrol (RSV) protects the endothelium upon high glucose (HG); however, the mechanisms underlying such protective effects are still debated. Here we identified key molecular players involved in the glycative/oxidative perturbations occurring in endothelial cells exposed to HG. In addition, we determined whether RSV essentially required SIRT1 to trigger adaptive responses in HG-challenged endothelial cells. We used primary human umbilical vein endothelial cells (HUVECs) undergoing a 24-h treatment with HG, with or without RSV and EX527 (i.e., SIRT1 inhibitor). We found that HG-induced glycative stress (GS) and oxidative stress (OS), by reducing SIRT1 activity, as well as by diminishing the efficiency of MG- and ROS-targeting protection. RSV totally abolished the HG-dependent cytotoxicity, and this was associated with SIRT1 upregulation, together with increased expression of GLO1, improved ROS-scavenging efficiency, and total suppression of HG-related GS and OS. Interestingly, RSV failed to induce effective response to HG cytotoxicity when EX527 was present, thus suggesting that the upregulation of SIRT1 is essential for RSV to activate the major antiglycative and antioxidative defense and avoid MG- and ROS-dependent molecular damages in HG environment.

## 1. Introduction

Chronic hyperglycemia, a distinctive feature of diabetes mellitus, is known to activate in tissues biochemical pathways leading to both glycative stress (GS) and oxidative stress (OS) [1]. Both GS and OS are thought to be critically involved in the onset of vascular complications, one of the most frequent and severe co-morbidities of diabetes mellitus [2,3,4,5,6]. GS is mainly due to the uncontrolled buildup of a highly reactive dicarbonyl glycolytic metabolite, methylglyoxal (MG) [7,8], whereas OS is caused by unrestrained accumulation of reactive oxygen species (ROS) [9]. If not promptly removed by dedicated antiglycative enzymes, such as glyoxalase 1 (GLO1) and glyoxalase 2 (GLO2), or by antioxidant enzymes, such as catalase (CAT), Cu/Zn-superoxide dismutase (SOD1), and Mn-superoxide dismutase (SOD2), MG and ROS can chemically attack key cellular macromolecules, thus leading to the production of oxidative damage and advanced glycation end-products (AGEs), respectively, resulting ultimately in cellular dysfunctions [1,7,8,10,11]. The catalytic dependence of GLO1 on glutathione (GSH) and the MG-driven glycation of mitochondrial proteins, along with the resulting ROS overproduction, tightly link GS and OS [5,7,12]. This link is strengthened by the fact that AGEs are often able to activate powerful ROS-generating pro-inflammatory pathways [7,13].

Given the importance of redox signaling and the pivotal role of AGEs in the pathogenesis of diabetes-related vascular complications, a great interest is rising in finding interventional strategies aimed at improving antioxidative efficiency and antiglycative defense in the endothelium exposed to high glucose (HG) [14,15,16,17].

Resveratrol (trans-3,5,4′-trihydroxystilbene; RSV), a plant-derived low molecular weight phytoalexin, is a nutraceutical agent through which redox-based pathologies (e.g., cardiovascular diseases, glucose intolerance, and insulin resistance) may be treated [18,19]. Some animal-based studies have shown anti-hyperglycemic properties of RSV, and patients with type 2 diabetes mellitus (T2DM) exhibited reduced oxidative stress, as well as improved insulin sensitivity and cardiovascular function after RSV treatment [20,21,22,23]. Moreover, RSV supplementation was demonstrated to ameliorate diabetes-related deterioration of systemic redox milieu and brain antioxidant capacity [24,25,26]. The beneficial bioeffects of RSV may arise from direct antioxidant properties and/or activation of endogenous self-defense mechanisms of the host cells [19]. The latter effects may be due to enzyme modification, direct upregulation of redox-responsive genes, and activation of sirtuin 1 (SIRT1), an important member of the family of NAD^+^-dependent class III deac(et)ylases [27]. As a key protein governing metabolic adaptation, DNA repair, cell survival, and oxidative stress response [28,29,30,31,32,33], SIRT1 is a potential target for interventions aimed at controlling hyperglycemia-associated impairments in metabolic functions and redox milieu. Interestingly, some of us have recently demonstrated that SIRT1 is required to control cellular glycative stress via regulation of the glyoxalase system [34]. Accordingly, Irshad and co-workers [35] have recently reported that RSV contributes to inhibit HG-induced dicarbonyl stress in human endothelial cells via upregulation of GLO1.

Considering all the above, it may be hypothesized that SIRT1 could represent the necessary mediator of the RSV-activated antiglycative and antioxidative protective action on the endothelial environment exposed to high glucose.

Despite the large number of data suggesting that RSV can protect the vascular environment against HG-induced dysfunctions, the underlying mechanisms at the molecular level, in terms of determinants and effectors, remain incompletely understood. In particular, the role of MG metabolism still needs to be elucidated. More specifically, it has to be ascertained whether RSV protects endothelial cells from HG-dependent cytotoxicity mostly via direct redox effects or through the modulation of the main endogenous antiglycative defense machinery. More importantly, the question as to whether RSV requires SIRT1 to elicit its cytoprotective effects in endothelial cells under HG condition is still unanswered.

On this basis, the aim of this work was to identify the molecular targets involved in the glycative perturbations occurring in human umbilical vein endothelial cells (HUVECs) exposed to HG, as well as to determine whether RSV is able to protect HUVECs from HG cytotoxicity via modulation of the major MG-targeting detoxification system. Last but not least, our study was aimed at establishing the role of SIRT1 in the RSV-evoked responses against HG challenge. Finally, based on the tight link between MG metabolism, redox milieu, and ROS scavenging, we extended our investigation to the major antioxidative enzymatic and non-enzymatic systems.

## 2. Materials and Methods

### 2.1. Antibodies

Abcam (Cambridge, UK) provided the following primary antibodies: anti-SIRT1 (ab7343; dil. 1:1000), anti-GLO1 (ab171121; dil. 1:1000), anti-SOD1 (ab16831; dil.1:1000), anti-SOD2 (ab86087; dil.1:1000), anti-CAT (ab16731; dil. 1:2000), and anti-β-actin (cat. ab8227; dil. 1:40000). The anti-argpyrimidine antibody (cat. AGE06B; dil. 1:1000) was provided by Nordic-MUbio (Susteren, The Netherlands). The HRP-conjugated goat anti-rabbit IgG secondary antibody (PI1000; dil. 1:1000) was purchased from Vector Laboratories (Burlingame, CA, USA). The peroxidase-conjugated anti-mouse secondary antibody (A9044; dil. 1:1000) was purchased from Sigma-Aldrich (Milan, Italy).

### 2.2. Cell Culture

Human umbilical vein endothelial cells (cat. C2519A, pooled donor) and the Clonetics^TM^ EGM^TM^-2 BulletKit^TM^ medium (cat. CC-3162) were purchased from Lonza (Basel, Switzerland). HUVECs were cultivated as recommended by the supplier in an incubator set at 37 °C, with 5% CO_2_ and saturated humidity. HUVECs were detached and sub-cultured when approx. 80% confluence was reached, and passages 3 or 4 were used for all experiments.

### 2.3. Cell Treatments

Two days after seeding HUVECs were incubated in the complete medium containing 5.5 mM (CTR) or 30.55 mM (HG) D-glucose (cat. G7021, Sigma-Aldrich, Milan, Italy) for 24 h, on the basis of existing literature [36]. As needed, RSV and EX527, a specific inhibitor of SIRT1 [37] (cat. R5010 and E7034, respectively, from Sigma-Aldrich, Milan, Italy), were administered to the cell culture. In particular, 48 h after seeding HUVECs were treated with different concentration of either RSV (range: 0–5 µM) or EX527 (range: 0–13.4 µM) for 24 h to evaluate cytotoxicity and SIRT1 expression. This preliminary experiment allowed us to determine the lowest effective concentrations (LECs) that were able to either up- or downregulate SIRT1. On this basis, all subsequent experiments were carried out by treating HUVECs upon 24-h HG with the LECs of RSV (5 µM) or RSV + EX527 (5 µM and 13.4 µM, respectively).

### 2.4. Cell Growth and Viability

Cells were seeded at a density of 2500 cells/cm^2^ and grown as specified above. Cell counting was performed by using the Trypan Blue staining method (cat. 15250-061, Life Technologies Italia, Monza, Italy) in a hemocytometer chamber, with three replicates.

### 2.5. RNA Extraction and Real-Time Quantitative Relative PCR (RT-PCR)

RNA was extracted from control and treated cells by using Ribospin kit (cat. 304-150), and the possible contaminant DNA was degraded by Riboclear plus (cat. 313-150) (all from GeneAll Biotechnology, Seoul, South Korea), following the supplier’s recommendations. The resulting RNA was used to obtain cDNA via reverse transcription (cat. NP100041, OriGene Technologies, Rockville, MD, USA). The cDNA (dil. 1:100) was used for PCR in an Applied Biosystems 7300 system (ThermoFisher Scientific, Rockford, IL, USA), using the TaqMan^®^ Gene Expression Master Mix (cat. 4369016, Applied Biosystems, Foster City, CA, USA) and TaqMan gene expression assays - FAM-MGB, according to the supplier’s instructions. The assay IDs used were: 18S-Hs99999901-S1, SIRT1-Hs01009006_m1, SOD1-Hs00533490_m1, SOD2-Hs00167309_m1, CAT-Hs00156308_m1, and GLO1-Hs00198702_m1. The amplification reaction was performed as follows: 2 min at 50 °C and 10 min at 95 °C, then 45 cycles of 15 s at 95 °C and 1 min at 60 °C. Gene expression was calculated by using the 2^−ΔΔCt^ method, using 18S as the reference mRNA and CTR sample as the calibrator [38]. The assays were performed with three replicates.

### 2.6. Total Protein Extraction and Western Blotting Analysis

Whole extracts from control and treated cells were obtained in RIPA buffer (cat. R0278, Sigma-Aldrich, Milan, Italy), which was supplemented with protease inhibitors (cat. P8340, Sigma-Aldrich, Milan, Italy) and phosphatase inhibitors I and III (cat. P2850 and P5726, respectively, from Sigma-Aldrich, Milan, Italy). After centrifugation at 16,000× *g* for 30 min at 4 °C, supernatants were assayed for total protein content, by using the BCA Protein Assay Kit and bovine serum albumin (BSA) as the standard (cat. PR23225, Euroclone, Milan, Italy). Denatured samples (10–20 μg) were run in triplicates on polyacrylamide gels (12%), and protein bands were transferred onto polyvinylidene difluoride (PVDF) membranes by electrophoretic transfer [39,40]. Non-specific binding sites were blocked at room temperature for 1 h with 5% (*w/v*) Blotting-Grade Blocker (cat. 170–6404, Bio-Rad Laboratories, Hercules, CA, USA), in Tris-buffer saline containing 0.05% (*v/v*) Tween-20 (cat. P5927, Sigma-Aldrich, Milan, Italy) (TBS-T). Membranes were incubated overnight with the primary antibodies diluted in TBS-T, and, then, with the peroxidase-conjugated secondary antibody for 2 h. The images of the specific immune complexes were revealed, acquired and analyzed by using Enhanced Chemi-luminescent Substrate Kit (cat. EMP001005, Euroclone, Milan, Italy), AllianceLD2 hardware (UVItec Limited, Cambridge, UK), and Total Lab TL120 software (TotalLab, Newcastle upon Tyne, UK). β-actin was used as the loading control for data normalization.

### 2.7. Measurement of SIRT1 Activity

Control and treated cells were lysed (20 mM Tris-Cl, pH 7.4, 135 mM NaCl, 1.5 mM MgCl_2_, 1 mM EGTA, 10% glycerol, 1% Nonidet P-40) and proteins were immunoprecipitated with anti-SIRT1-conjugated protein A agarose beads (cat. ab7343 and ab193254, respectively, Abcam, Cambridge, UK). Briefly, the agarose beads were mixed with primary antibody for 4 h at 4 °C (15 µg of anti-SIRT1 antibody/mL of beads). The mixture was then centrifuged and washed with the aforementioned lysis buffer several times. After the last wash, cell lysates (2 mg of total proteins) were incubated overnight with the antibody-beads mixture. The immunecomplexes were centrifuged and washed, and the immunoprecipitates were directly used for the SIRT1 activity assay kit (cat. ab156065, Abcam, Cambridge, UK), according to supplier’s instructions [16,41]. The reaction mixtures were prepared by mixing 17 µL _dd_H_2_O, 5 µL fluoro-substrate peptide, 5 µL NAD^+^, 5 µl developer, and 13 µL test samples. Fluorescence (ex. 355 nm, em. 460 nm) was detected for 70 min, with 2 min intervals, in a Victor3 microplate reader (PerkinElmer, Waltham, MA, USA). The assays were performed with three replicates.

### 2.8. Cell Lysis Extraction for Enzymatic Activity

Control and treated cells (6–8 × 10^6^ cells/mL) were lysed in either 100mM KH_2_PO_4_, 1.5 mM dithiotreitol (DTT), and 1mM ethylenediaminetetraacetic acid (EDTA) (pH 7) extraction buffer (for GLO1 enzyme activity) or 100mM KH_2_PO_4_ (pH 7), 0.1% Triton X-100 (for total SOD, SOD2, and CAT). Samples were centrifuged at 16000× *g* for 30 min at 4 °C, and the supernatants were used for assessments of enzymatic activities and total protein concentration (cat.500-0006, BioRad, Hercules, CA, USA), with BSA used as the standard. All readings were carried out in triplicates or quadruplicates, using a Lamba25 UV–VIS spectrophotometer (PerkinElmer, Waltham, MA, USA).

### 2.9. Measurement of GLO1 Specific Activity

The GLO1 (EC 4.4.1.5) activity was measured at 240 nm, by recording the appearance of (R)-S-lactoylglutathione, as described by Mannervik and co-workers [42]. The reaction mixture contained 1 mM glutathione (cat. G4251, Sigma-Aldrich, Milan, Italy) and 2 mM methylglyoxal (cat. M0252, Sigma-Aldrich, Milan, Italy). One unit was defined as 1 µmol of (R)-S-lactoylglutathione formed/min at 25 °C.

### 2.10. Measurement of CAT Activity

The CAT (EC 1.11.1.6) activity was assayed by monitoring the decomposition of 10 mM H_2_O_2_ (cat. 21676-3, Sigma-Aldrich, Milan, Italy) at 240 nm, as described by Aebi [43]. One unit was defined as 1 µmol of H_2_O_2_ reduced/min at 25 °C.

### 2.11. Measurement of SOD Activity

Total SOD (EC 1.15.1.1) activity was assayed in 50 mM NaHCO_3_ buffer (pH 10.2), containing 25 mM EDTA and 0.1 M epinephrine bitartrate (cat. E4375, Sigma-Aldrich, Milan, Italy). SOD ability to inhibit the epinephrine auto-oxidation was monitored at 480 nm, according to Sun and Zigman [44]. One unit was defined as the amount of enzyme required to halve the rate of epinephrine autoxidation at 30 °C. After assessing tSOD specific activity, lysates were treated with 1 mM KCN (cat. 207810, Sigma-Aldrich, Milan, Italy) to inactivate SOD1 [45], and measure the SOD2 activity. SOD1 activity was calculated by subtraction (tSOD-SOD2).

### 2.12. Apoptosis/Necrosis Assay

Cell death was determined using an IncuCyte Live Cell Analysis System (Essen Bioscience, Ann Arbor, MI, USA). Briefly, HUVECs were seeded (2500 cells/cm^2^) and cultured in 96-well black plates, with three replicates. After 48 h, cells were treated as described in the cell treatment sub-section (2.3), in presence of Alexa Fluor 488 annexin V (cat. 10040-02, SouthernBiotech, Birmingham, AL, USA) and propidium iodide (PI) (cat. P4170, Sigma-Aldrich, Milan, Italy) for 24 h. Final imaging data (4 × magnification) were acquired and analyzed. Annexin V^+^/PI^−^ cells were considered as early apoptotic, annexin V^+^/PI^+^ were counted as late apoptotic/necrotic, and annexin V^−^/PI^−^ cells were considered as alive [46].

### 2.13. TBARS Assay

Lipid peroxidation is one of the early biochemical event occurring upon high glucose stress [47]. Accordingly, lipid peroxidation-derived molecules were found to be increased in several organs of diabetes in vivo models [48], as well as in cells and plasma of diabetic patients [49,50]. In our study, the levels of thiobarbituric acid reactive substances (TBARS) were determined according to the method described by Yagi [51], using the TBARS Assay kit (cat. 10009055, Cayman Chemical, Ann Arbor, MI, USA). Briefly, control and treated cells were extracted in 37.5 μL PBS and the resulting samples were mixed with 1 volume of SDS. Then, color reagent (2 mL) was added to each sample in triplicates. The reaction mixtures were incubated for one hour in boiling water and centrifuged at 1600× *g* for 10 min at 4 °C. Supernatants were read at 532 nm by a Lambda25 spectrophotometer (PerkinElmer, Waltham, MA, USA). TBARS concentrations of unknown samples were interpolated on a linear calibration curve that was obtained from pure malondialdehyde (MDA)-containing reactions (0–50 μM).

### 2.14. Scanning Electron Microscopy (SEM)

Scanning electron microscopy (SEM) was carried out on HUVECs left to adhere overnight on coverslips that were pre-coated with poly-L-lysine and fixed with 2% glutaraldehyde (Electron Microscopy Sciences, Hatfield, PA, USA) in PBS for 30 min. The coverslips were briefly rinsed with PBS and water and, then, dehydrated in ethanol solutions 30–50–70–90% in H_2_O, and three times in ethanol 100%, for 10 min each. For hexamethyldisilazane (HMDS) drying, the samples were immersed for 3 min in 100% HMDS (Electron Microscopy Sciences) and, then, the excess HMDS was blotted away by filter paper. Samples were transferred to a desiccator for 25 min to avoid water contamination and mounted on stubs sputter-coated with chromium in a Quorumtech Q 150T ES Turbo chromium sputter. Then, samples were detected by a Zeiss Gemini SEM 500 [52].

### 2.15. Glutathione Assay

Total glutathione (tGSH) and glutathione disulfide (GSSG) levels were determined by using a glutathione assay kit (cat. 703002, Cayman Chemical) [53]. Briefly, control and treated cells were lysed in the MES buffer provided by the kit (1.5 × 10^7^ cells/mL), and centrifuged at 10,000× *g* for 15 min, at 4 °C. Cell lysates were deproteinized with 5% (*w/v*) metaphosphoric acid (cat. 239275, Sigma-Aldrich, Milan, Italy) and centrifuged at 4000× *g* for 5 min, as recommended by the supplier. In order to evaluate the abundance of GSSG, GSH in the samples was first derivatized with 2-vinylpyridine (cat. 132292, Sigma-Aldrich, Milan, Italy). Protein-free samples (50 µL) and the assay cocktail (150 µL) were loaded in a 96-well plate, with three replicates. Absorbance at 405 nm was followed for 30 min, with 5 min intervals, by using a Victor3 microplate reader (PerkinElmer, Waltham, MA, USA). tGSH and GSSG concentrations of experimental samples were interpolated on calibration curves that were obtained from reactions containing either pure GSH or pure GSSG standards (0–16 μM tGSH or 0–8 μM GSSG). Results were given as GSSG over GSH ratio.

### 2.16. Statistics

Data were expressed as means and S.E.M. of N independent experiments (as reported in the figures’ captions). Microsoft Excel 2007, Statistica 10, and GraphPad Prism 6 packages were used for data processing and visualization. The homogeneity of variances and normal distribution were verified using Bartlett’s and Shapiro–Wilk tests. One-way ANOVA and post-hoc Tukey tests were used to evaluate statistically significant differences. Differences were considered as statistically significant with *p* < 0.05.

## 3. Results

### 3.1. RSV Upregulated/EX527 Downregulated SIRT1 with no Cytotoxic Effects

**Figure 1:**
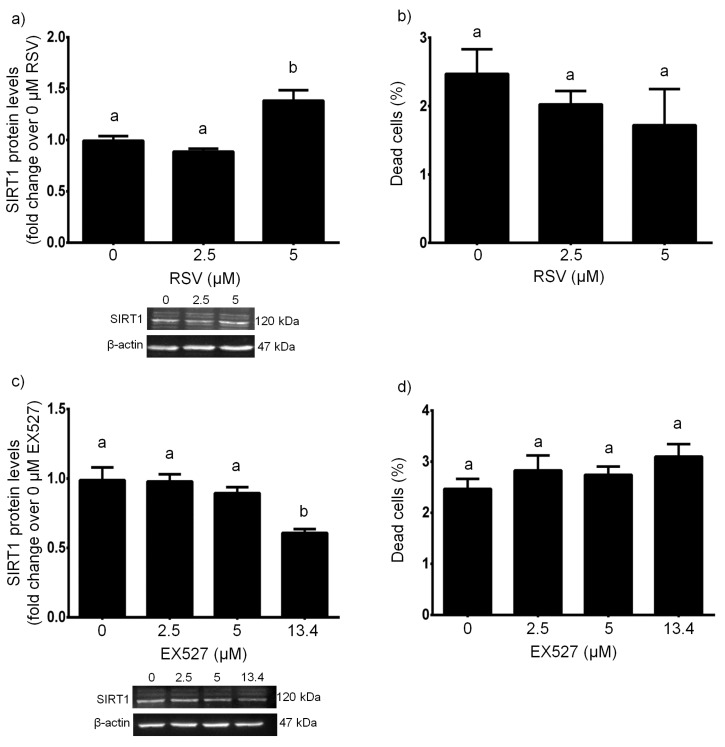
Effects of a 24-h treatment with either resveratrol (**a**,**b**) or EX527 (**c**,**d**) on sirtuin 1 protein levels (**a**,**c**) and cell death (**b**,**d**) in HUVECs. Representative blots are also shown (**a**,**c**). Data are expressed as means and S.E.M. Abbreviations: RSV resveratrol, SIRT1 sirtuin 1. Means sharing the same letter are not significantly different from each other (one-way ANOVA and Tukey’s multiple comparisons test): Panel (**a**): a vs. b, *p* < 0.01 (*N* = 4); Panel (**b**): (*N* = 5); Panel (**c**): a vs. b, *p* < 0.05 (*N* = 4); Panel (**d**): (*N* = 4–9).

Our ANOVA-based analyses revealed that, when treated with RSV at different concentrations for 24 h, HUVECs showed statistically significant changes in SIRT1 protein expression (*F* = 15.46, *p* < 0.01). In particular, 5 µM RSV upregulated SIRT1 protein expression, with respect to untreated cells (*p* < 0.01, 5 µM vs. 0 µM), whereas the treatment with 2.5 µM RSV was not sufficient to change SIRT1 protein levels (*p* > 0.05, 2.5 µM vs. 0 µM) (Figure 1a). We did not find any statistically significant changes in cell viability (*F* = 0.9537, *p* > 0.05), and this was confirmed by post-hoc multiple comparisons when all RSV concentrations were tested (*p* > 0.05 for all comparisons) (Figure 1b).

The 24-h treatment with EX527 at different concentrations significantly altered SIRT1 protein expression (*F* = 9.161, *p* < 0.01). In detail, 13.4 µM EX527 downregulated SIRT1 protein expression (*p* < 0.05, 13.4 µM vs. 0 µM), whereas the treatments with 2.5 µM and 5 µM EX527 could not affect SIRT1 protein levels (*p* > 0.05, 2.5 µM vs. 0 µM and 5 µM vs. 0 µM) (Figure 1c). We could not detect any statistically significant changes in cell viability (*F* = 1.527, *p* > 0.05), and this was confirmed post-hoc multiple comparisons when all EX527 concentrations were tested (*p* > 0.05 for all comparisons) (Figure 1d).

### 3.2. SIRT1 Mediated the Cytoprotective Effect of RSV on HG-Challenged HUVECs

**Figure 2:**
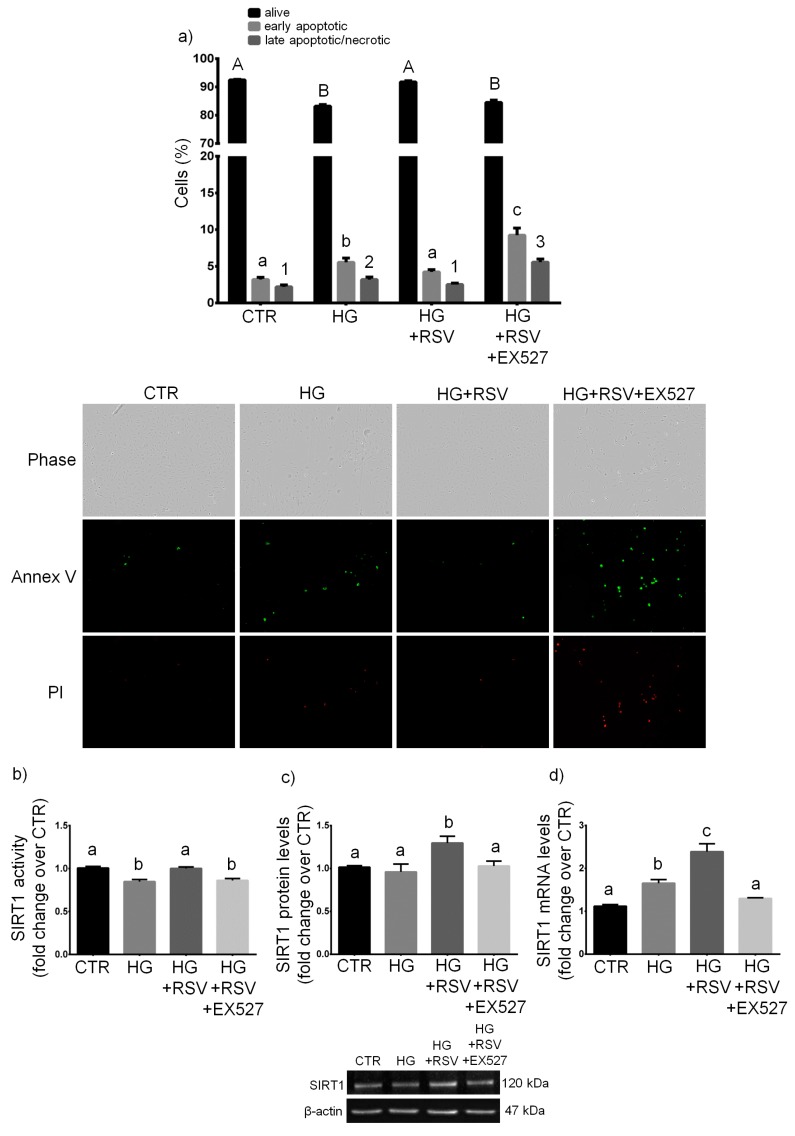
Effects of a 24-h treatment with either resveratrol alone (5 µM) or resveratrol + EX527 (5 µM and 13.4 µM, respectively) combination on viability, apoptosis, and necrosis (**a**), as well as on enzymatic activity (**b**), protein level (**c**), and transcript amount (**d**) of sirtuin 1 in high glucose-challenged HUVECs. Representative microphotographs (10 ×) and western blots are also shown (**a**,**c** respectively). Data are expressed as means and S.E.M. Abbreviations: Annex V—annexin V; CTR—vehicle; HG—high glucose; HG + RSV—high glucose + resveratrol; HG + RSV + EX527—high glucose + resveratrol + EX527; SIRT1—sirtuin 1; PI—propidium iodide. Means sharing the same letter (or number) are not significantly different from each other (one-way ANOVA and Tukey’s multiple comparisons test): Panel (**a**): A vs. B, *p* < 0.001; a vs. b, *p* < 0.05; b vs. c, *p* < 0.001; a vs. c, *p* < 0.001; 1 vs. 2, *p* < 0.05; 1 vs. 3, *p* < 0.001; 2 vs. 3, *p* < 0.001 (*N* = 5). Panel (**b**): a vs. b, *p* < 0.01 (*N* = 4). Panel (**c**): a vs. b, *p* < 0.05 (*N* = 4). Panel (**d**): a vs. b, *p* < 0.05; b vs. c, *p* < 0.001; a vs. c, *p* < 0.001 (*N* = 5).

HUVECs were treated for 24 h with vehicle alone, HG, HG + RSV, or HG + RSV + EX527, and cell growth, necrosis and apoptosis were assayed, along with mRNA, protein and activity of SIRT1. As expected, the experimental treatments caused statistically significant changes in viability (*F* = 357.8, *p* < 0.001), early apoptosis (*F* = 95.73, *p* < 0.001), and late apoptosis/necrosis (*F* = 114.6, *p* < 0.001). The one-way ANOVA also revealed that the experimental conditions altered significantly SIRT1 enzymatic activity (*F* = 17.11, *p* < 0.001), as well as SIRT1 transcript (*F* = 29.43, *p* < 0.001) and protein (*F* = 4.947, *p* < 0.05). In particular, the 24-h treatment with HG increased the number of apoptotic and necrotic cells (*p* < 0.05, HG vs. CTR), and diminished the number of viable cells (*p* < 0.001, HG vs. CTR) (Figure 2a). Such a detrimental effect was associated with decreased SIRT1 activity (*p* < 0.01, HG vs. CTR; Figure 2b), that was mirrored by an increase in SIRT1 transcript level (*p* < 0.05, HG vs. CTR; Figure 2c), even though no change in SIRT1 protein level was detected (*p* > 0.05, HG vs. CTR; Figure 2d).

RSV totally abolished the cytotoxic effects of HG and completely restored the cell viability (*p* > 0.05, CTR vs. HG + RSV). Such beneficial effect was associated with a complete return of SIRT1 activity to levels observed in controls (*p* > 0.05, HG + RSV vs. CTR; Figure 2b), along with strongly increased levels of SIRT1 transcript (*p* < 0.001, HG + RSV vs. HG; Figure 2d) and protein (*p* < 0.05, HG + RSV vs. HG; Figure 2c).

As expected, EX527 (i.e., a specific inhibitor of SIRT1) abolished the RSV-induced increase in SIRT1 activity (*p* > 0.05, HG + RSV + EX vs. HG; Figure 2b) and decrease in protein (*p* > 0.05 HG + RSV + EX vs. HG; Figure 2c). In addition, in the presence of inhibitor, SIRT1 transcriptional expression was repressed to levels observed in untreated cells (*p* > 0.05 HG + RSV + EX vs. CTR; Figure 2d). Such downregulation was associated to a failure for RSV to elicit cytoprotective effects in HG-challenged HUVECs, as shown by the diminished cell viability (*p* < 0.001, HG + RSV + EX527 vs. HG + RSV) and increased cell death (both apoptotic and necrotic; *p* < 0.001, HG + RSV + EX527 vs. HG + RSV), with respect to cells treated with the combination HG + RSV (Figure 2a).

**Figure 3:**
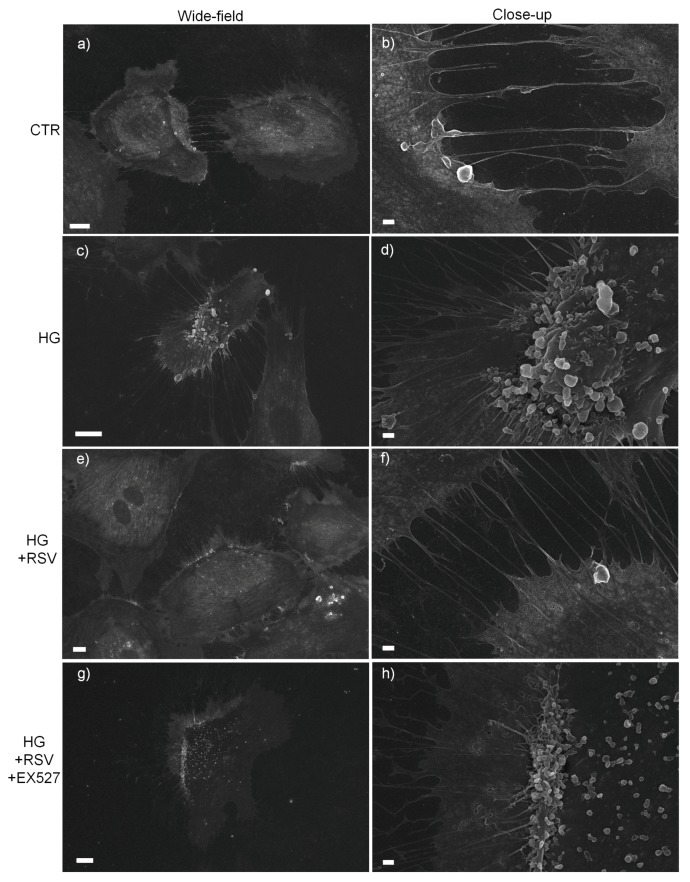
Effect of a 24-h treatment with either resveratrol alone (5 µM) or resveratrol + EX527 (5 µM and 13.4 µM, respectively) on cell membrane response of high glucose-challenged HUVECs. Representative scanning electron microscope images are shown. Bars: 10 µm (**a**,**c**,**e**,**g**), 1 µm (**b**,**d**,**f**,**h**). Abbreviations: CTR—vehicle; HG—high glucose; HG + RSV—high glucose + resveratrol; HG + RSV + EX527—high glucose + resveratrol + EX527.

Given the association between the high glucose-dependent activation of endothelial cells and the release of extracellular vesicles and changes in membrane dynamics [54,55,56], we investigated by scanning electron microscopy (SEM) whether RSV was able to revert the HG-induced changes in cell membrane dynamics in a SIRT1-dependent fashion. SEM-based analysis revealed markedly increased extracellular vesicle (EVs)-presenting cells upon HG (Figure 3c,d), in contrast to what was seen in normal glucose (Figure 3a,b). Interestingly, SEM observations showed that RSV ameliorated such HG-induced membrane re-arrangements (Figure 3e,f), whereas such effect was abolished by EX527 (Figure 3g,h).

### 3.3. RSV Protected HUVECs from HG-Induced Oxidative and Dicarbonyl Stress via SIRT1 Upregulation

**Figure 4:**
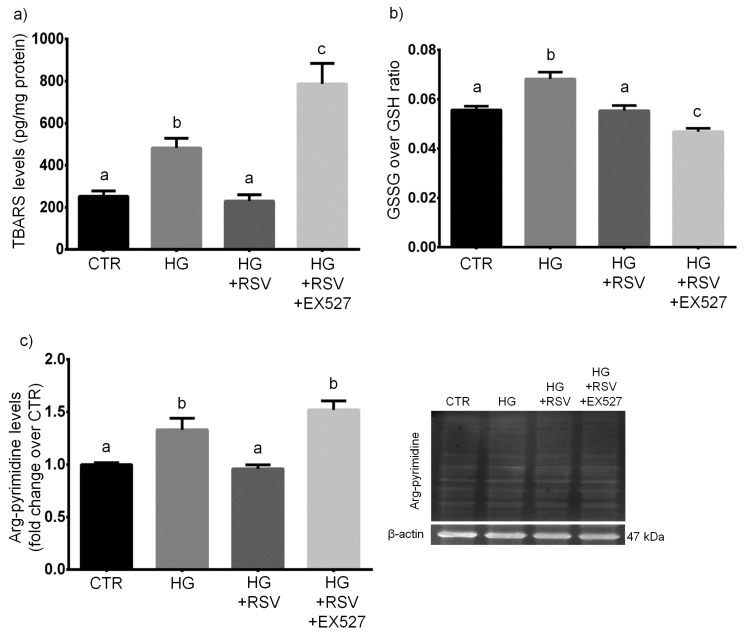
Effects of a 24-h treatment with either resveratrol alone (5 µM) or resveratrol + EX527 (5 µM and 13.4 µM, respectively) combination on TBARS levels (**a**), glutathione redox balance (**b**), and arg-pyrimidine concentration (**c**) in high glucose-challenged HUVECs. Representative blots are also shown (**c**). Data are expressed as means and S.E.M. Abbreviations: CTR—vehicle; HG—high glucose; HG + RSV—high glucose + resveratrol; HG + RSV + EX527—high glucose + resveratrol + EX527; GSH—reduced glutathione; GSSG—glutathione disulfide; TBARS—thiobarbituric acid-reactive substances. Means sharing the same letter are not significantly different from each other (one-way ANOVA and Tukey’s multiple comparisons test): Panel (**a**): a vs. b, *p* < 0.05; b vs. c, *p* < 0.05; a vs. c, *p* < 0.001 (*N* = 4). Panel (**b**): a vs. b, *p* < 0.001; b vs. c, *p* < 0.001; a vs. c, *p* < 0.05 (*N* = 8). Panel (**c**): a vs. b, *p* < 0.05 (*N* = 4).

HUVECs were treated with vehicle alone, HG, HG + RSV, or HG + RSV + EX527 for 24 h, and redox−/glycation-related parameters were assayed. The one-way ANOVA showed that the redox balance of glutathione, along with the levels of peroxidative damage and glycative damage were altered by the experimental treatments (*F* = 19.37, *p* < 0.001, for GSSG/GSH; *F* = 20.28, *p* < 0.001, for TBARS; *F* = 13.68, *p* < 0.001, for arg-pyrimidine). In detail, our analyses revealed that, as expected, the 24-h treatment with HG induced oxidative and glycative stress, as shown by the augmented levels of TBARS and arg-pyrimidine (*p* < 0.05, HG vs. CTR; Figure 4a,c). In addition, we found that HG caused a redox imbalance, as shown by an increased GSSG over GSH ratio (*p* < 0.001, HG vs. CTR; Figure 4b).

RSV totally reverted the pro-oxidant and pro-glycation effects of the 24-h HG treatment, as shown by the return of the levels of TBARS and arg-pyrimidine to control values (*p* > 0.05, HG + RSV vs. CTR; Figure 4a,c). Moreover, RSV totally canceled the HG-related pro-oxidant shift in the redox balance of glutathione (*p* > 0.05, HG + RSV vs. CTR; Figure 4b).

EX527 inhibited the RSV-dependent protection against HG-induced pro-glycation effect, as shown by the similar levels of arg-pyrimidine levels found in HG + RSV + EX and HG (*p* > 0.05, HG + RSV + EX vs. HG; Figure 4c). In addition, in the presence of EX527 the HG-induced increase in TBARS levels was even worsened, as compared to that observed in HUVECs treated with HG alone (*p* < 0.05, HG + RSV + EX vs. HG; Figure 4a). Unexpectedly, the GSSG/GSH ratio resulted to be further lowered by EX527, with respect to cells treated with HG + RSV (*p* < 0.05, HG + RSV + EX vs. HG + RSV; Figure 4b).

### 3.4. RSV Enhanced ROS Scavenging Efficiency in HG-Challenged HUVECs via SIRT1 Upregulation

Since the RSV-induced response of HUVECs to HG in terms of glutathione redox status seemed not to depend on SIRT1, we extended our study to the major GSH-independent antioxidant enzymatic systems, namely the superoxide dismutases (SODs) and the catalase (CAT).

HUVECs were treated with vehicle alone, HG, HG + RSV, or HG + RSV + EX527 for 24 h, and ROS-scavenging enzymes were measured at mRNA, protein and activity levels. The statistical analysis of data revealed that specific activities of SODs and CAT were significantly altered by the experimental treatments (*F* = 8.424, *p* < 0.01, for SOD1; *F* = 30.69, *p* < 0.001, for SOD2; *F* = 51.53, *p* < 0.001, for CAT). One-way ANOVA showed also that the experimental treatments changed significantly protein levels of both SODs (*F* = 15.70, *p* < 0.001, for SOD1; *F* = 12.22, *p* < 0.001, for SOD2), whereas no statistically significant change was detected for CAT (*F* = 0.5497, *p* > 0.05). The ANOVA revealed also a significant main effect of the experimental treatments (*F* = 24.72, *p* < 0.001) on the ratio between CAT and tSOD activities. Finally, one-way ANOVA revealed that mRNA levels of SODs and CAT were changed significantly as a result of the experimental treatments (*F* = 24.35, *p* < 0.001, for SOD1; *F* = 20.28, *p* < 0.001, for SOD2; *F* = 21.81, *p* < 0.001, for CAT).

**Figure 5:**
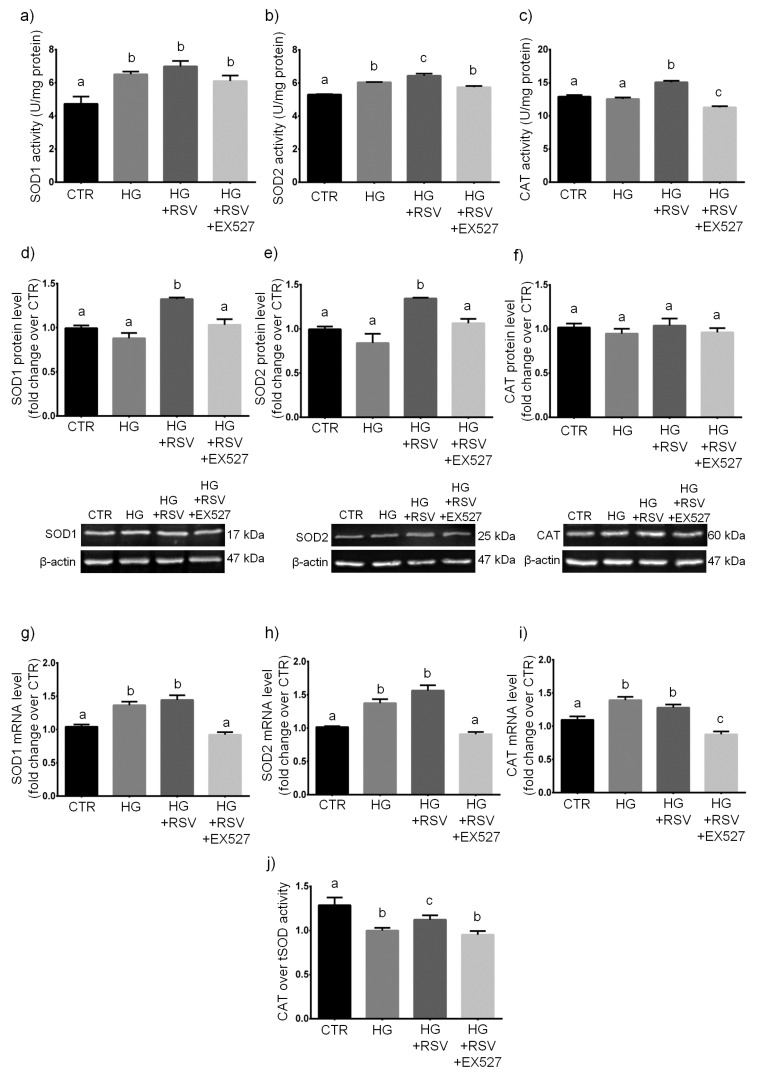
Effects of a 24-h treatment with either resveratrol alone (5 µM) or resveratrol+EX527 (5 µM and 13.4 µM, respectively) combination on the enzymatic activity (**a**–**c**), protein levels (**d**–**f**), and mRNA content (**g**–**i**) of superoxide dismutase 1 (**a**,**d**,**g**), superoxide dismutase 2 (**b**,**e**,**h**), and catalase (**c**,**f**,**i**) in high glucose-challenged HUVECs. The catalase over total superoxide dismutase ratio was also shown (**j**), as well as representative blots (**d**–**f**). Data are expressed as means and S.E.M. Abbreviations: CTR—vehicle; HG—high glucose; HG + RSV—high glucose + resveratrol; HG + RSV + EX527—high glucose + resveratrol + EX527; SOD1—superoxide dismutase 1; SOD2—superoxide dismutase 2; tSOD—total superoxide dismutase; CAT—catalase. Means sharing the same letter are not significantly different from each other (one-way ANOVA and Tukey’s multiple comparisons test): Panel (**a**): a vs. b, *p* < 0.05 (*N* = 3–4). Panel (**b**): a vs. b, *p* < 0.05; b vs. c, *p* < 0.05; a vs. c, *p* < 0.001 (*N* = 3–4). Panel (**c**): a vs. b, *p* < 0.001; b vs. c, *p* < 0.001; a vs. c, *p* < 0.05 (*N* = 3–4). Panel (**d**): a vs. b, *p* <.01 (*N* = 4). Panel (**e**): a vs. b, *p* < 0.05 (*N* = 4). Panel (**f**): (*N* = 4). Panel (**g**): a vs. b, *p* < 0.01 (*N* = 5). Panel (**h**): a vs. b, *p* < 0.05 (*N* = 3–5). Panel (**i**): a vs. b, *p* < 0.05; a vs. c, *p* < 0.05; b vs. c, *p* < 0.001 (*N* = 5). Panel (**j**): a vs. b, *p* < 0.001; b vs. c, *p* < 0.05; a vs. c, *p* < 0.05 (*N* = 3–4).

In detail, our analyses revealed that the 24-h HG treatment caused an increase of the specific activity of both SOD1 and SOD2 (*p* < 0.05, HG vs. CTR; Figure 5a,b), and this was accompanied by a similar increase in their mRNAs (*p* < 0.01 and *p* < 0.05, HG vs. CTR; Figure 5g,h). A similar HG-induced elevation of mRNA was observed for CAT (*p* < 0.05, HG vs. CTR; Figure 5i). However, CAT activity was found to be unchanged in HG, as compared to untreated cells (*p* > 0.05, HG vs. CTR; Figure 5c). None of the three enzymes was modulated by HG at translational level (*p* > 0.05, HG vs. CTR; Figure 5d–f). In addition, since the ratio of specific activities of coupled antioxidant enzymes much better reflects the status of the antioxidative enzymatic defense than each activity alone [57,58,59], we calculated the CAT/tSOD ratio, which is far more indicative of the overall ROS-targeting efficiency. Our data showed that HG strongly reduced the CAT over tSOD ratio, with respect to untreated cells (*p* < 0.001, HG vs. CTR; Figure 5j).

Our findings indicated that RSV partially recovered the HG-induced drop in ROS-targeting efficiency (*p* < 0.05, HG + RSV vs. HG; Figure 5j). In the presence of RSV, the HG-induced transcriptional upregulation of SOD1, SOD2, and CAT was unaffected (*p* > 0.05, HG + RSV vs. HG; Figure 5g–i), however protein levels of SODs were greater than those observed in cells treated with HG alone (*p* < 0.05, HG + RSV vs. HG; Figure 5d,e), whereas CAT protein amount remained unchanged (*p* > 0.05, HG + RSV vs. HG; Figure 5f). Intriguingly, only the activities of SOD2 and CAT resulted to be improved when HUVECs were treated with the HG + RSV combination, as compared to cells treated with HG alone (*p* < 0.05 and *p* < 0.001, HG + RSV vs. HG; Figure 5b,c). More interestingly, our calculations revealed that RSV partially inhibited the HG-induced drop in CAT/tSOD ratio (*p* < 0.05, HG + RSV vs. HG; Figure 5j).

In the presence of EX527, the transcriptional upregulation of SODs that was promoted by HG+RSV was totally abolished, as demonstrated by the similar levels of both mRNA between cells treated with HG + RSV + EX and untreated cells (*p* > 0.05, HG + RSV + EX vs. CTR; Figure 5g,h). Surprisingly, HUVECs treated with HG + RSV + EX527 exhibited CAT mRNA levels that were even lower than those detected in untreated cells (*p* < 0.001, HG + RSV + EX vs. CTR; Figure 5i). In addition, EX527 blocked the RSV-induced upregulation of SOD1 and SOD2 at protein level (*p* < 0.05, HG + RSV + EX vs. HG + RSV; Figure 5d,e). Finally, by co-incubating HG-challenged cells with EX527, the RSV-induced improvement of SOD2 activity was also inhibited (*p* < 0.05, HG + RSV + EX vs. HG + RSV; Figure 5b). Surprisingly, our data also indicated that the RSV-induced partial recovery of the HG-dependent drop in CAT/tSOD ratio was lost in EX527-treated HUVECs, as shown by the very similar CAT over tSOD ratios found in cells treated with either HG+RSV+EX or HG alone (*p* > 0.05, HG + RSV + EX vs. HG; Figure 5j).

### 3.5. RSV Improved MG Scavenging Activity in HG-Challenged HUVECs via SIRT1 Upregulation

**Figure 6:**
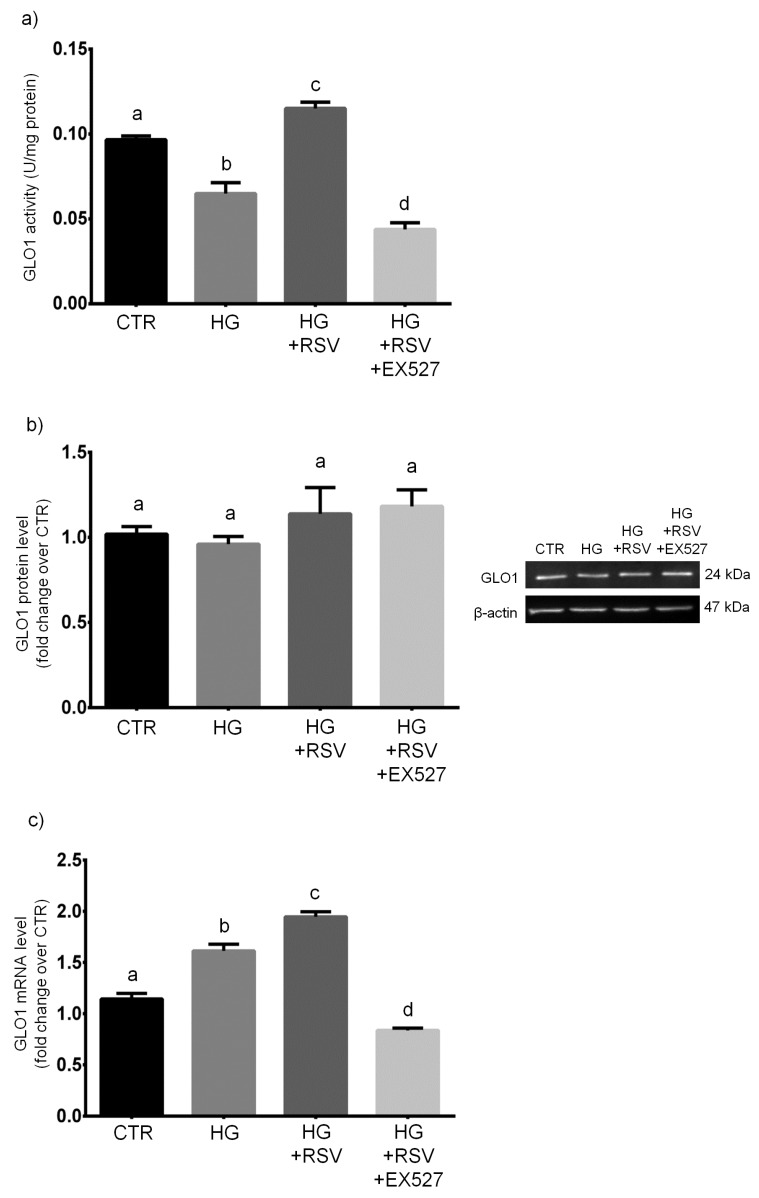
Effects of a 24-h treatment with either resveratrol alone (5 µM) or resveratrol+EX527 (5 µM and 13.4 µM, respectively) combination on the enzymatic activity (**a**), protein levels (**b**), as well as mRNA content (**c**) of GLO1 in high glucose-challenged HUVECs. Representative blots are also shown (**b**). Data are expressed as means and S.E.M. Abbreviations: CTR—vehicle; HG—high glucose; HG + RSV—high glucose + resveratrol; HG + RSV + EX527—high glucose + resveratrol + EX527; GLO1—glyoxalase 1. Means sharing the same letter are not significantly different from each other (one-way ANOVA and Tukey’s multiple comparisons test): Panel (**a**): a vs. b, *p* < 0.01; b vs. c, *p* < 0.001; a vs. c, *p* < 0.05; b vs. d, *p* < 0.05; a vs. d, *p* < 0.001; c vs. d, *p* < 0.001 (*N* = 4). Panel (**b**): (*N* = 4). Panel (**c**): a vs. b, *p* < 0.001; b vs. c, *p* < 0.01; a vs. c, *p* < 0.001; a vs. d, *p* < 0.01; b vs. d, *p* < 0.001; c vs. d, *p* < 0.001(*N* = 5).

HUVECs were treated with vehicle alone, HG, HG + RSV, or HG + RSV + EX527 for 24 h, and the MG-scavenging GLO1 was measured at mRNA, protein and activity levels. One-way ANOVA analysis showed that the specific activity of glyoxalase 1, along with GLO1 mRNA levels, was significantly altered by the experimental treatments (*F* = 53.67, *p* < 0.001 and *F* = 95.21, *p* < 0.001, respectively), whereas the protein amount of GLO1 was unaffected by the treatments (*F* = 1.135, *p* > 0.05). In particular, we found that the 24-h HG treatment caused a dramatic decrease of the specific activity of GLO1, the major MG-targeting enzyme (*p* < 0.01, HG vs. CTR; Figure 6a). This event was mirrored by an increase in GLO1 mRNAs (*p* < 0.001, HG vs. CTR; Figure 6c), even though unaffected levels of GLO1 protein were detected in HG-challenged HUVECs (*p* > 0.05, HG vs. CTR; Figure 6b).

Interestingly, RSV overboosted GLO1 activity, which resulted to be even higher than that detected in untreated cells (*p* < 0.001, HG + RSV vs. HG; Figure 6a), and this was paralleled by augmented levels of GLO1 transcript (*p* < 0.01, HG + RSV vs. HG; Figure 6c), even though the amount of GLO1 protein remained unchanged (*p* > 0.05, HG + RSV vs. HG; Figure 6b).

Surprisingly, the presence of EX527 totally inhibited the RSV-induced improvement of GLO1 specific activity, which resulted to be even lower than that measured in untreated cells (*p* < 0.001, HG + RSV + EX vs. CTR; Figure 6a). Such an event was accompanied by a similar decrease of GLO1 mRNA (*p* < 0.01, HG + RSV + EX vs. CTR; Figure 6c), but not by a parallel drop in GLO1 protein amount, that remained at the same level detected in HUVECs challenged with HG + RSV (*p* > 0.05, HG + RSV + EX vs. HG + RSV; Figure 6b).

## 4. Discussion

Despite the increasing interest on how RSV can protect endothelial cells against HG-induced biomolecular impairments, much is still to be discovered in terms of determinants and effectors, with particular focus on cellular responses involving the modulation of ROS- and MG-targeting systems. Most importantly, the question as to whether RSV requires SIRT1 as a crucial mediator to elicit cytoprotective effects in endothelial cells upon HG still remains debated.

Our experiments clearly showed that, as expected, HG elicited a cytotoxic effect in human primary endothelial cells. Indeed, we found that the 24-h incubation with 30.55 mM high glucose induced cell death (both apoptotic and necrotic) in HUVECs, as previously shown by many other researchers [14,15,36,60,61,62,63,64,65]. SEM data confirmed that HG induced clear changes in membrane dynamics and massive production of extracellular vesicles, which are known to be related to high glucose-dependent activation of endothelial cells [54,55,56]. In this context, the presence of some antioxidant enzymes in HUVEC-derived microvesicles have been documented. In particular, Soleti et al. [66] established that HUVECs have a complete antioxidant machinery and that their microvesicles included a specific group of functional enzymes mainly involved in detoxification of both superoxide anion and hydrogen peroxide. We observed that the cytotoxic effect of HG was associated with evidence of oxidative and glycative cellular stress, as shown by the markedly increased peroxidative and glycative damage markers (i.e., TBARS and arg-pyrimidine, respectively). HG induced also an increase in GSSG/GSH ratio, which points to a pro-oxidant shift in the redox balance of glutathione. Upon redox stress, an important defense is provided by the enzymatic couple CAT/SOD, which ensures a rapid flux in the removal process of the redox couple O_2_^−^/H_2_O_2_ [45,57,58]. Our assays revealed a prominent reduction in the CAT/SOD ratio upon HG, which is suggestive of an impairment of the O_2_^−^/H_2_O_2_ detoxification process. Similarly, we found that HG induced a strong decrease of the specific activity of the GLO1, and this, together with the lower availability of GSH, represents a clue of a seriously impaired MG-scavenging enzymatic system. Among the most important regulators of the ROS-targeting enzymatic defense is SIRT1, which is a key NAD^+^-dependent deac(et)ylase whose dysregulation often underlies unsuccessful responses to redox-based stressors [28,29,30,31,32,33]. In coherence with other works [67,68], we found that HG induced a decrease in SIRT1 activity. According to existing literature, this could represent one of the major determinants of the oxidative stress commonly observed in hyperglycemic states [69]. However, we found that the decline in SIRT1 activity was paralleled by an increase in the mRNA coding for sirtuin 1, and this may point to a possible attempt of a transcriptional adaptive response occurring in HUVECs exposed to a HG environment. This was also observed for SODs, CAT, and GLO1, which were all transcriptionally upregulated by HG. However, the corresponding proteins were not found to be increased similarly. This suggests that the attempted adaptive response was ineffective, and this might be due to an uncoupling between transcription regulation and translation processes. Such phenomena could be related to the extensive oxidative and glycative damage occurring in the HG condition, which is known to impair severely protein functioning, as well as the efficiency of intracellular signaling [1,13,70,71].

Our findings are in accordance with other reports that showed a reduction of SIRT1 activity, along with the occurrence of glycative/oxidative damage and loss of antiglycative and antioxidant capacity in endothelial cells exposed to a HG environment [14,15,35,36,61,63,67,68]. This confirmed the reliability of our HG model, which is somehow representative of the glucose levels reported by the criteria for the diagnosis of diabetes [72].

Our experiments revealed that RSV effectively protected HG-challenged human primary endothelial cells from HG cytotoxicity and inhibited the HG-induced release of extracellular vesicles. This was associated with the activation of a SIRT1-dependent multiple response that prevented the strong HG-dependent membrane activation, as well as the oxidative and dicarbonyl damage. In detail, RSV totally reverted the pro-apoptotic and pro-oxidant effects of HG, along with the pro-oxidant shift in the redox balance of glutathione. This was probably achieved by preventing the loss of efficiency in the removal process of superoxide anion and hydrogen peroxide, as the HG-dependent decline in the CAT/SOD ratio was partially reverted by co-incubation with RSV. Our findings are in accordance with previous data which showed increased GSH content and SOD activity in human umbilical vein endothelial cells after RSV treatment [73]. In addition, in arterial endothelial cells, RSV has been found to upregulate at transcriptional level γ-glutamylcysteine synthetase, the rate limiting enzyme in GSH biosynthesis [74]. Interestingly, our results showed that SOD2 is more responsive to RSV, as compared to SOD1, and this is in accordance with existing literature [75,76]. In fact, Ungvari and co-workers [77] found evidence of an RSV-induced increase in mitochondrial antioxidant capacity in coronary arterial endothelial cells, and this was achieved via the upregulation of SOD2, the crucial ROS scavenging enzyme in the mitochondrial matrix. Other researchers showed that RSV activates SOD2 and upregulates mitochondrial biogenesis, thus lowering the mitochondrial load during respiration [78,79,80]. In our opinion, the particular responsiveness of SOD2 to RSV is worth further investigation. Hence, our future experiments will investigate more in depth the possible involvement of mitochondria in the RSV-induced rewiring of antiglycative and antioxidant response, extending our investigations to the role of mitochondria-centered sirtuins (SIRT3-5). Interestingly, resveratrol increased both mRNA and specific activity, but not protein levels, of catalase, thus pointing to a possible regulation of CAT expression through post-transcriptional and post-translational modifications, which might be activated only in presence of the redox imbalance induced by HG.

Redox stress and dicarbonyl stress are tightly linked, and this is partly due to the catalytic dependence of GLO1 on GSH availability, as well as to the MG-driven glycation of mitochondrial proteins, which both may lead to ROS overproduction and oxidative stress [5,7,12]. We here showed that RSV impeded the HG-induced impairment of glyoxalase-mediated MG-scavenging activity, and inhibited the increase of MG-dependent glycative damage upon HG. In fact, the HG-dependent marked decrease of GLO1 specific activity was abolished by RSV co-treatment. More in detail, the GLO1 enzymatic activity in HUVECs treated with HG+RSV was found to be even higher than that detected in untreated control cells. This, together with the higher availability of GSH, is an evidence of an improvement in the efficiency of MG-scavenging enzymatic system occurring in RSV-treated cells. This was further confirmed by the strong decrease of MG-dependent protein damage observed in HUVECs incubated with RSV+HG, as compared to cells treated with HG alone. Taking into account two very recent reports, one from some of us [34], in which the SIRT1 was proven to be essential for RSV to activate GLO1-based response to MG in oocytes, and one from Irshad and colleagues [35], who reported that human aortal endothelial cells upon HG were protected against GLO1 activity loss and overproduction of MG if treated with RSV+hesperetin, we hypothesized that SIRT1 could be required for RSV to upregulate GLO1-based protection against glycative damage in HG-challenged HUVECs. On this basis, we demonstrated that when the RSV-induced upregulation of SIRT1 was inhibited by EX527, resveratrol was not able to promote any adaptive response to HG in terms of GLO1 expression and activity, and this was confirmed by the higher levels of MG-dependent damage we found in HUVECs that were treated with HG+RSV+EX527, as compared to cells incubated with HG+RSV. Similarly, when SIRT1 was inhibited, the RSV-dependent recovery of ROS scavenging efficiency was lost, and this was true especially at the level of SOD2 and CAT. This was further confirmed by the greater peroxidative damage we detected in HUVECs that were treated with HG+RSV+EX527, with respect to cells incubated with HG+RSV. Some researchers suggested that RSV may modulate the expression of catalase in cultured human endothelial cells [81,82], and SIRT1 was suspected to mediate that effect [83,84]. Similarly, the upregulation of SODs by RSV was also associated with a likely activation of SIRT1 signaling [77,82]. Interestingly, we observed that the EX527-induced repression of the antioxidant and antiglycative responses to HG triggered by RSV in HUVECs appeared to occur at transcriptional level, as shown by the strongly downregulated mRNA of SODs, CAT, and GLO1. In particular, EX527 lowered mRNA levels of CAT and GLO1 to less than that observed in untreated cells. This may point to a possible SIRT1-dependent modulatory action of RSV on the antioxidant defense systems through the regulation of transcriptional factors, such as the nuclear factor erythroid 2-related factor 2 (NRF2) or the nuclear factor-κB (NF-κB). RSV has been linked to regulation of NRF2-Keap1 signaling [85,86]. The pioneering work of Ungvari et al. [74] revealed that resveratrol is able to confer endothelial protective effects by activating NRF2, both in vitro and in vivo. More recently, the treatment of HaCaT keratinocytes with RSV resulted in enhanced nuclear localization of NRF2, along with increased activities of antioxidant enzymes, which protected these cells from UV radiation-induced apoptosis [87]. In addition, Csiszár et al. [88] demonstrated that upon treatment with resveratrol encapsulated in fusogenic liposomes, aged cerebromicrovascular endothelial cells showed activated NRF2, significantly decreased cellular production of reactive oxygen species, as well as inhibited apoptosis.

NRF2 might be not the only transcriptional factor involved in the RSV-SIRT1-induced regulation of redox- and glycation-related metabolism. In fact, GLO1 can be transcriptionally regulated by NF-κB signaling, as reported by Antognelli and Talesa [89]. In addition, NF-κB has been shown to be actively involved in the upregulation of CAT in response to oxidative stress [90].

Our future works will attempt to clarify the role of NF-κB or NRF2 in the cytoprotective effects of RSV on HG-challenged human endothelial cells.

Taken together, our results show that when the RSV-promoted upregulation of SIRT1 was repressed by EX527, all the molecular and biochemical adaptations induced by RSV appeared to be inhibited, and HUVECs were no longer protected against HG-dependent cell death. This suggests that the cytoprotective effect of RSV on HG-challenged HUVECs is strictly dependent on SIRT1 upregulation.

Surprisingly, SIRT1 was not essential to the RSV-induced restoration of the redox homeostasis of glutathione, as indicated by the lower GSSG/GSH ratio measured in cells treated with HG + RSV + EX527, as compared to cells kept in HG + RSV. This may suggest that the inhibition of SIRT1, along with the HG challenge, possibly triggered the activation of de novo synthesis of glutathione and/or the improvement of glutathione recycling. Further experiments are needed to confirm such a hypothesis.

## 5. Conclusions

**Figure 7:**
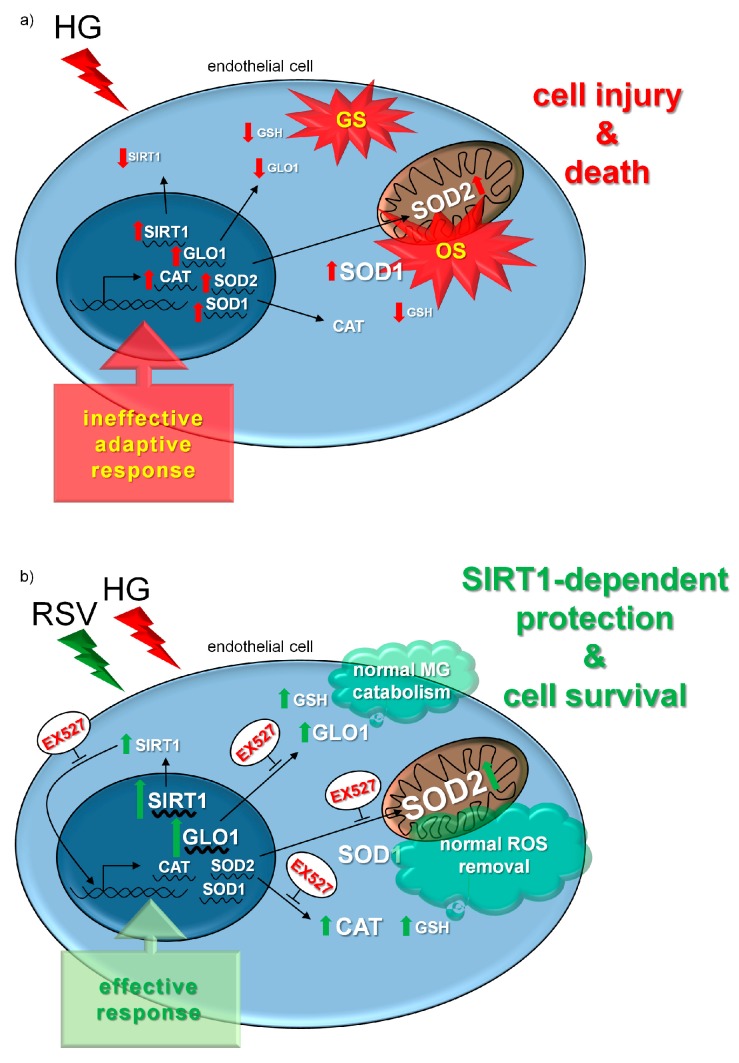
Synoptical interpretation of the experimental results. A 24-h treatment with HG induced both GS and OS in HUVECs by reducing SIRT1 activity, as well as by diminishing the efficiency of MG- and ROS-targeting defense. An attempt of compensation occurred at transcriptional level, however this was ineffective, as HG promoted apoptotic and necrotic cell death (**a**). The cytotoxic effects of HG were abolished by the co-treatment with RSV, and this was associated with transcriptionally-regulated restoration of SIRT1 activity, together with increased expression of GLO1, improved ROS-scavenging efficiency, and total suppression of HG-related GS and OS. The RSV-induced effective response to HG cytotoxicity was abolished by the specific inhibitor of SIRT1 (i.e., EX527), and this started at the level of mRNA transcription, thus suggesting that the upregulation of SIRT1 was essential for RSV to activate the major antioxidative and antiglycative defense, and avoid ROS- and MG-dependent molecular damages in a high glucose environment (**b**). Abbreviations: CAT—catalase; HG—high glucose; GLO1—glyoxalase 1; GS—glycative stress; GSH—reduced glutathione; OS—oxidative stress; RSV—resveratrol; SIRT1—sirtuin 1; SOD1—superoxide dismutase 1; SOD2—superoxide dismutase 2.

In this study, we demonstrated that SIRT1 upregulation is essential for RSV to exert protective effects on human primary endothelial cells that were challenged with high glucose. In addition, we established that the RSV-induced upregulation of SIRT1 is critically required to activate the major antioxidative and antiglycative defenses and avoid ROS- and MG-dependent molecular damages in a high glucose environment (Figure 7). In particular, the ability of RSV to protect endothelial cells, not just because of its unspecific direct antioxidant properties, but through the activation of the SIRT1-GLO1 pathway, may be of great relevance. In fact, many experimental and clinical studies confirmed that GLO1 inducers and/or dicarbonyl scavengers represent potential effective agents to reverse or limit diabetes-related complications [70,91,92,93,94].
